# Experimental Simulation of the Effects of an Initial Antibiotic Treatment on a Subsequent Treatment after Initial Therapy Failure

**DOI:** 10.3390/antibiotics3010049

**Published:** 2014-02-17

**Authors:** Yanfang Feng, Nadine Händel, Marnix H. P. de Groot, Stanley Brul, Constance Schultsz, Benno H. ter Kuile

**Affiliations:** 1 Laboratory for Molecular Biology and Microbial Food Safety, Swammerdam Institute for Life Sciences, University of Amsterdam, 1098 XH Amsterdam, The Netherlands; E-Mails: Y.Feng2@uva.nl (Y.F.); N.Handel@uva.nl (N.H.); marnixdegroot@me.com (M.H.P.G.); S.brul@uva.nl (S.B.); 2 Department of Global Health-Amsterdam Institute for Global Health and Development and Department of Medical Microbiology, Academic Medical Center, University of Amsterdam, 1105 AZ Amsterdam, The Netherlands; E-Mail: schultsz@gmail.com; 3 Netherlands Food and Consumer Product Safety Authority, Office for Risk Assessment, 3511 GG Utrecht, The Netherlands

**Keywords:** therapy failure, resistance development, amoxicillin, cefotaxime

## Abstract

Therapy failure of empirical antibiotic treatments prescribed by primary care physicians occurs commonly. The effect of such a treatment on the susceptibility to second line antimicrobial drugs is unknown. Resistance to amoxicillin was rapidly induced or selected in *E. coli* at concentrations expected in the patient’s body. Strains with reduced susceptibility outcompeted the wild-type whenever antibiotics were present, even in low concentrations that did not affect the growth rates of both strains. Exposure of *E. coli* to amoxicillin caused moderate resistance to cefotaxime. The combined evidence suggests that initial treatment by amoxicillin has a negative effect on subsequent therapy with beta-lactam antibiotics.

## 1. Introduction

Bacterial infections are often initially treated empirically with first-line antibiotics, such as amoxicillin. Because of the global prevalence of antibiotic resistance, the failure of the first therapy has become a frequent event [1]. Subsequent treatment is required in such cases to eradicate the enduring infection. It is not clear, however, how the initial antibiotic therapy influences the follow-up treatment. Clinical observations suggest that there is an effect of the first on the later treatments, but the microbial physiology that can explain this effect is not understood at present.

Within the patient, it is not possible to distinguish between certain cells becoming resistant and an already existing resistant subpopulation becoming dominant at the infection site during antimicrobial therapy. Therefore, the initial question to be addressed is whether under conditions that can be expected to occur in a patient during treatment using a given dosing schedule, pathogens that are initially susceptible to the antibiotic applied can become resistant. The ensuing question is whether a strain that has developed resistance can hamper the follow-up treatment, due to simultaneously acquired reduced susceptibility to the next antimicrobial drug, in particular when a more potent drug of the same class is chosen.

The *de novo* emergence of resistance as a result of adaptation and mutations due to exposure to antibiotics is well-documented [2,3,4,5]. When resistance is not acquired through horizontal gene transfer, amoxicillin resistance in *Escherichia coli* is mostly caused by the induction of AmpC beta-lactamase [6,7,8]. Exposure to non-lethal levels of antibiotics induces a complex series of adaptations at the expression and cellular level affecting metabolism, regulation, virulence, DNA repair and stress response [6,9,10]. Those changes might result in cross-resistance to other antibiotics that are eligible for subsequent treatments, especially if they have similar mechanisms [11]. Even if the increase of resistance may appear limited, at the infection site, such effects could determine the difference between the elimination and survival of pathogens.

When antibiotic treatment is applied, another possible development in an infection site is the selection of a subpopulation that is already moderately to highly resistant. In fact, the survival of cells of a less susceptible subpopulation is a digital event: either it happens or it does not. According to the mutant selection window hypothesis, selection of resistant cells only occurs when the drug concentration exceeds the MIC (Minimal Inhibitory Concentration) of the susceptible cells (MICsusc), but is below that of the resistant variants (MPC) (Mutant Prevention Concentration) [12,13]. However, concentrations lower than MICsusc can also effectively select for resistant strains, as long as the fitness cost of resistance does not exceed the metabolic advantages [14,15]. That selection for resistance at low antibiotic levels occurs in an *in vivo* model was shown in rabbits infected with *Staphylococcus aureus* [16].

Levels of the antibiotic are not constant within the patient during a therapy. Only when the medicine is delivered intravenously can constant blood levels be expected. Typically, oral therapy involves the patient taking a dose at more or less regular intervals for some time, while the kidneys or liver remove the antibiotic after it has reached the bloodstream. In addition, the antibiotic may not penetrate well to the infection site, reducing the exposure of the pathogens even further [17,18]. Concentrations at which the selection for resistance takes place may therefore be encountered under a variety of conditions. Pre-existing mutations can be selected at high concentrations, but *de novo* mutations and adaptation at the expression level occur mostly when levels are low between the administrations of the doses [19]. These conditions can be simulated in the laboratory and the insights thus obtained used to improve treatment strategies.

The aim of this study is to simulate *in vitro* the outcome of a situation in which an initial amoxicillin treatment fails to cure an *E. coli* infection, since the drug concentration attained at the infection site is sub-lethal. By documenting the development of resistance as an effect of this event and the selection of strains that have become moderately resistant by this simulated treatment, the effects of a failed initial treatment on subsequent antibiotic therapy can be envisaged. The outcome of this *in vitro* study using *E. coli* as a model organism suggests that an initial amoxicillin treatment of an amoxicillin susceptible strain can negatively influence the outcome of continued amoxicillin treatment or a follow-up therapy with the third-generation cephalosporin cefotaxime. Even though cefotaxime is a beta-lactam antibiotic just as amoxicillin, it is still commonly prescribed in The Netherlands for follow-up treatment, as it is considered more potent, and while more than 40% of the isolates from general practice are resistant to amoxicillin, only 3%–6% are resistant to cefotaxime. The research presented below puts the effectiveness of this practice into doubt.

## 2. Results

**Figure 1:**
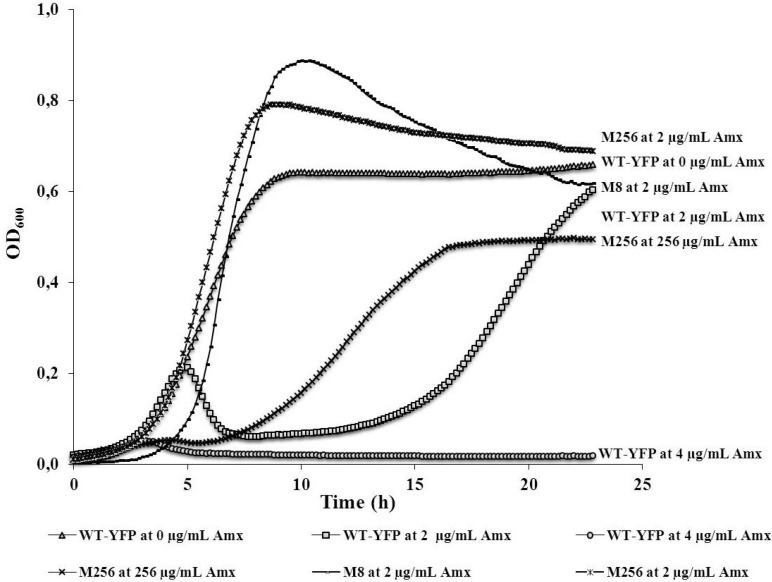
Representative growth curves of variants of *E. coli* MG1655 at different concentrations of amoxicillin. WT-YFP (wild-type-yellow fluorescent protein), the amoxicillin-susceptible variant (MIC 4 µg/mL), with genes coding for a yellow fluorescent protein and chloramphenicol resistance. M256, the strain derived from *E. coli* WT MG1655 by growing it at stepwise increasing amoxicillin concentrations. M8, WT-YFP after five days of growth at 2 µg/mL (MIC 32 µg/mL).

To illustrate the effect of amoxicillin on the growth of *E. coli* MG1655 wild-type, cultures were compared at zero, two and 4 µg/mL of this antibiotic (Figure 1). Growth was almost completely inhibited at 4 µg/mL. At 2 µg/mL, the initial growth rate equaled to that of the control, but the culture started to collapse after approximately 5 h. This suggests that most cells died as the cell wall disintegrated. It seems that afterwards, some of the cells that remained alive grew out, and after 23 h, a density was reached nearly identical to that of the control. This two-stage growth curve indicates that the rapid adaptation that allows cultures of *E. coli* to withstand amoxicillin levels close to the MIC is caused in part by the survival of a small subpopulation when the majority of the cells succumb. In a similar manner, growth was followed for the same strain that was made resistant by exposure to 2 µg/mL amoxicillin for five days (indicated as M8), or by exposure to step-wise increasing concentrations of amoxicillin [5] (indicated as M256) at the highest concentration that allowed growth, 256 µg/mL. At this concentration of amoxicillin, this highly-resistant strain had a lower growth rate, but reached a final density of about 75% of the control. At 2 µg/mL amoxicillin, the two adapted strains grew at the same rate as they did in the absence of antibiotics, indicating that no residual effects remained.

**Figure 2:**
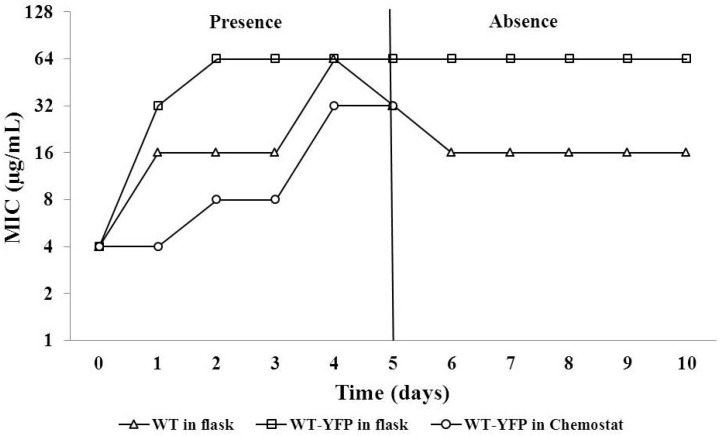
The increase of the MIC during growth of *E. coli* in the presence of 2 µg/mL amoxicillin for five days and subsequently in absence for another five days. WT, *E. coli* MG 1655. WT-YFP, WT with genes coding for chloramphenicol resistance and a yellow fluorescent protein. In the chemostat (D = 0.3 h^−1^), a pulse of amoxicillin reaching maximally 2 µg/mL in the culture vessel was given every 8 h.

To assess whether amoxicillin-susceptible *E.* *coli* could become resistant due to exposure to levels of amoxicillin below the MIC, the increase of the MIC was measured during growth at 2 µg/mL amoxicillin (Figure 2). In the presence of 2 µg/mL amoxicillin, the MICs of the wild-type (WT) and WT-YFP strains rapidly increased from 4 µg/mL to 16 or 32 µg/mL within 24-hour exposure, and eventually reached 32 or 64 µg/mL after another 4 days of culture. The WT-YFP yellow fluorescent protein) strain became resistant slightly faster. This acquired resistance remained or decreased by only a factor of two during growth in the absence of amoxicillin for the following five days.

In order to mimic carbon and energy limited growth and exposure to fluctuating drug concentrations, as might take place in the blood of a patient, a chemostat culture of WT was grown at a specific growth rate (D) of 0.3 h^−1^ and maximally 2 µg/mL amoxicillin. To simulate the usual treatment regimen of three oral doses per day over five days, the antibiotic was pumped in for one hour, reaching the maximum concentration, followed by 7 h, during which the drug was steadily diluted. Under these conditions, *E. coli* builds up resistance gradually (Figure 2). The MIC was elevated by a factor of two after every 1–2 days. Still, the final MIC of 32 µg/mL is considered clinically resistant according to the EUCAST (The European Committee on Antimicrobial Susceptibility Testing) system [20].

The irreversible nature of the increase in MIC suggests that a mutation is involved, not only adaptation at the expression level. To verify this, the promoter of the *ampC* lactamase gene, which is known to be involved in amoxicillin resistance [6], was sequenced to detect relevant mutations for all daily samples (Table 1). The WT flask culture first developed a mutation weakening its attenuator on the third day, followed by a mutation in the Pribnow box that optimizes the promoter function [8] on the following day. The WT-YFP flask culture only acquired the same Pribnow box mutation, but did so already on the second day of exposure. The other mutation was not observed. In the chemostat, however, there was no mutation detected throughout the exposure period, suggesting that adaptations at the expression level were sufficient to induce considerable resistance. The observations described above suggest that a typical five-day treatment of amoxicillin can induce resistance to a level that hampers further treatment, should this be necessary.

**Table 1:** Mutations in the promoter region of the *E. coli* *ampC* lactamase gene. Mutations in the promoter region of the *ampC* lactamase gene of WT and WT-YFP strains cultivated in flasks and WT-YFP cultivated in a chemostat vessel (see Figure 2). Each data point represents two sequenced PCR reactions from separate colonies from a plated-out stabilite sampled at the indicated day. Only in one case, Day 5 of the WT in a shaking flask, did the outcome of the two colonies differ. The strains obtained after five days are used for further experimentation and subsequently indicated as M8 and M8-YFP.

Time (days)	WT in flask		WT-YFP in flask		WT-YFP in chemostat	
	Position	Mutations	Position	Mutations	Position	Mutations
**0**	none	none	none	none	none	none
**1**	none	none	none	none	none	none
**2**	none	none	4377037	C-->T	none	none
**3**	4376996	G-->T	4377037	C-->T	none	none
**4**	4377037	C-->T	4377037	C-->T	none	none
**5**	4376996	G-->T	4377037	C-->T	none	none
	4377037	C-->T	4377037			

**Figure 3:**
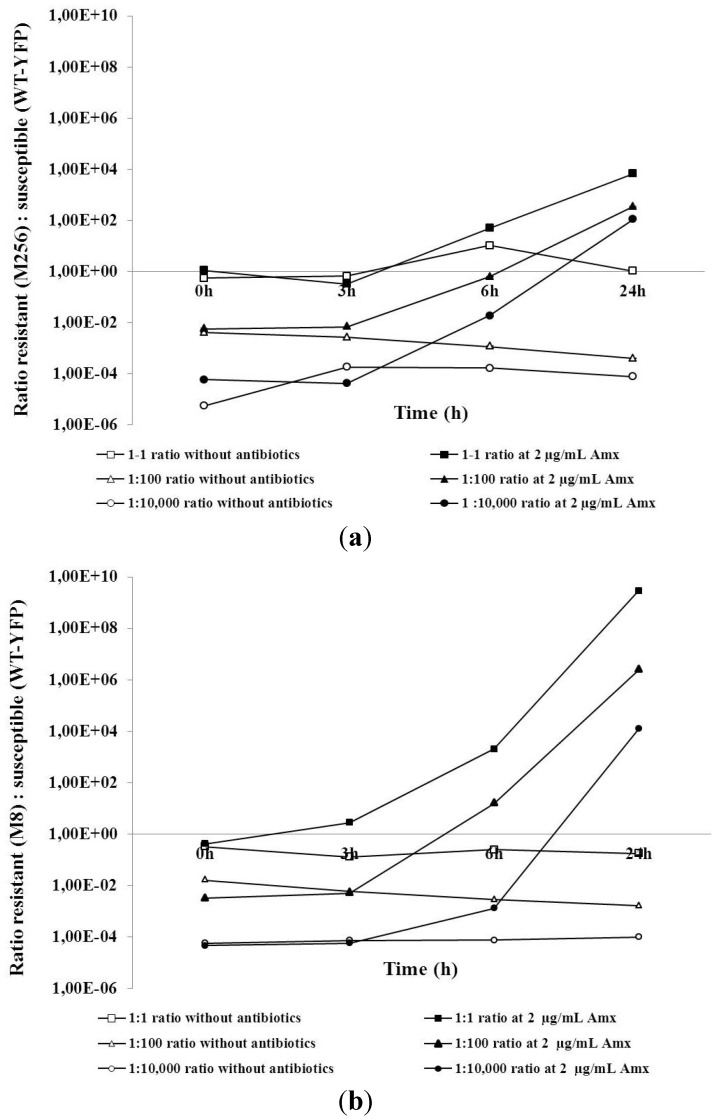
Competition experiments between M8 (**a**) or M256 (**b**) and WT-YFP with different initial ratios (1:1, 1:100 and 1:10,000) in the absence or presence of 2 µg/mL amoxicillin.

In order to explore whether *E. coli* cells that acquired resistance can cause treatment failure in follow-up therapy, competition experiments in a co-culture were carried out between wild-type (WT-YFP) and cells made moderately resistant (M8) or highly resistant (M256), as described above. Two sets of growth competition experiments were performed. In the first, the ratios between fully susceptible WT-YFP and the highly amoxicillin-resistant strain, M256, during growth at different amoxicillin levels were followed (Figure 3a). The second focused on the WT-YFP and the mildly resistant M8 strain (Figure 3b). The resistant and susceptible strains were mixed together in starting ratios of approximately 1:1, 100:1 or 10,000:1 and grown in medium containing 2 µg/mL amoxicillin or the same medium without antibiotics. Each line of Figure 3 represents a separate competition experiment in which changes in the ratio were monitored at zero, three, six and 24 h. In the absence of antibiotics, the ratios between the different strains remained basically unchanged, while the culture density increased by approximately a factor of 1,000. In the presence of amoxicillin, the resistant strains overgrew the susceptible. The change in the ratio was independent of the initial ratio. Changes were more drastic when M8 was competing with WT-YFP than in the case of WT-YFP and M256, indicating that at these low levels of amoxicillin, the moderately resistant strain had more advantage than the highly resistant one. Very similar results were obtained at 1 µg/mL amoxicillin (data not shown).

**Figure 4:**
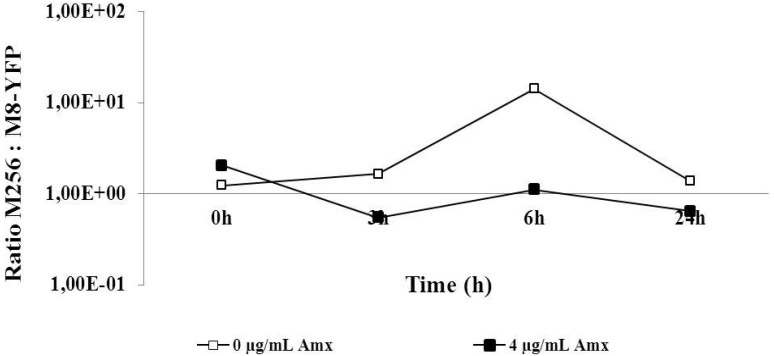
Competition experiment between M256 and M8 (see Figure 1) in an initial ratio of 1:1 and exposed to zero or 4 µg/mL amoxicillin.

Since the moderately resistant M8 strain was more effective than the highly resistant M256 in outcompeting the sensitive strain, the ability of M8-YFP, made moderately resistant against amoxicillin in the same way as the M8 strain, to outgrow M256 was explored. The ratios in the co-culture were basically constant during growth. In the absence of antibiotics, the M8-YFP strains seemed to have initially a marginal advantage, if at all. The disadvantage at 4 µg/mL amoxicillin was also minute or absent. Given that the most drastic change in the ratio was a factor of 10, compared to up to 10^10^ in the other experiments, the expected outcompeting of M256 by M8-YFP was effectively not observed (Figure 4).

**Figure 5:**
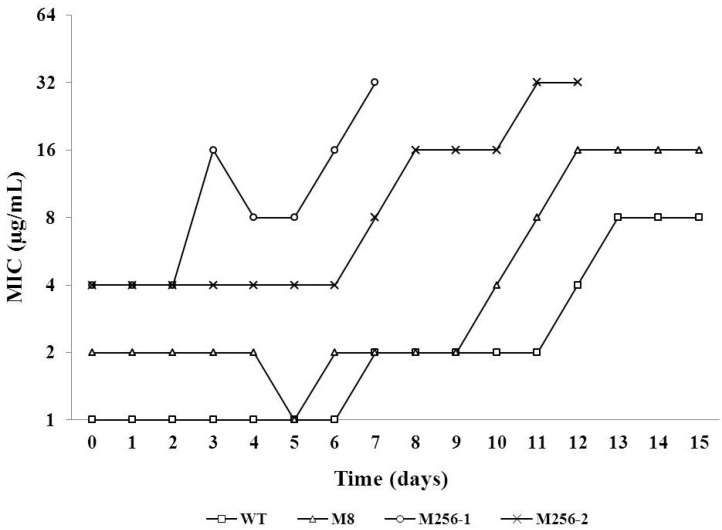
The MICs as a function of time during the adaptation of *E. coli* strains to stepwise increasing concentrations of cefotaxime, starting at 0.06 µg/mL for WT (MIC = 1 µg/mL) and WT 5a (see Figure 1; MIC = 2 µg/mL) and at 0.5 µg/mL for the two duplicate strains of M256, indicated as M256-1 and M256-2.

To evaluate the effects of previous amoxicillin exposure on the efficacy of subsequent cefotaxime treatment, the potential of adaptation to cefotaxime was compared among WT, M8 and M256. The M256’s initial MIC for cefotaxime exceeded that of the WT by a factor of four; that of M8, by a factor of two. The cultures of the two biological duplicates of the M256 strain both collapsed during the adaptation, one on the eight day, the other on the thirteenth day (Figure 5). However, the adjustment of WT and M8 to growth in the presence cefotaxime progressed smoothly till the fifteenth day. The MICs for cefotaxime of all tested strains were increased by three two-fold steps during adaptation.

To assess whether the overexpressed beta-lactamase of the M256 strain is further induced during the development of cefotaxime resistance, the beta-lactamase activity was measured before and after adaptation to cefotaxime (Table 2). Before adaptation, the M8 and WT strain presented almost the same level of beta-lactamase activity, while M256 exhibited an activity more than 200 times higher than WT and M8. After adaptation, the beta-lactamase activities of M8 and WT remained on the same level as prior to adaptation, but the enzyme activity of M256 was negligible, indicating that *E. coli*’s development of resistance to cefotaxime is less likely to be caused by overexpression of beta-lactamase and furthermore suggesting that cefotaxime does not induce an increase of beta-lactamase activity. As a result, the amoxicillin resistant M256 strain started out somewhat more resistant to cefotaxime, as well, and reached higher levels of cefotaxime resistance than the amoxicillin sensitive strains before collapsing when the beta-lactamase activity decreased.

**Table 2:** Specific activities of the beta-lactamase of the WT, M8 and M256 strains (see Figure 1), before and after adaptation to cefotaxime (see Figure 5). The results are presented as the means and standard deviations of three biological duplicates. For each biological replicate, two independent measurements were performed.

Strains	Beta-lactamase activity	
	Before adaptation	After adaptation
**Supernatant**	0	0
**WT**	21.6 ± 2.9	23.3 ± 4.5
**M8**	21.4 ± 3.3	17.2 ± 1.3
**M256**	491.3 ± 3.6	3.6 ± 1.8

## 3. Discussion

It is not uncommon in clinical practice that different antibiotics might be employed successively to cure a single infection after initial therapy failure [1]. In case amoxicillin is not sufficiently effective, cefotaxime, a third generation cephalosporin, is sometimes applied for the follow-up treatment. Therefore, the effect of the initial amoxicillin treatment on the effectiveness of cefotaxime was addressed. In The Netherlands, resistance against cefotaxime is far less common than resistance against amoxicillin. As cefotaxime is in addition considered more potent, it is often used for follow-up treatment after amoxicillin failed to cure the infection, even though both belong to the beta-lactam class. In establishing this practice, the effects of the first treatment on the effectiveness of the subsequent one were not considered. We have explored *in vitro* how preceding exposure to amoxicillin influences the susceptibility of a culture to amoxicillin itself or cefotaxime as an example of another beta-lactam antibiotic. Extrapolated to medical practice, this implicates that an initial amoxicillin therapy may negatively influence the clinical outcome of subsequent treatments with the same or another beta-lactam antibiotic.

Failure of an initial amoxicillin treatment might be encountered clinically in those cases that the drug concentration reaching the infection site is lower than the optimal level [21]. In an infection site, the bacterial population is likely to be heterogeneous and consisting of cells and strains possessing a range of MICs. The actual antibiotic concentration at the infection site might be lethal for some strains, while others survive, even if they are not defined as clinically resistant [21]. This notion can be illustrated by the result that most wild-type cells died initially when exposed to 2 µg/mL amoxicillin, while some cells grew out after 18 h (Figure 1). These surviving cells could rapidly develop moderate, but long-lasting, resistance as the exposure to amoxicillin continued (Figure 2), in agreement with the large number of physiological and genetic changes that are induced by sublethal levels of antibiotics [4,6]. One example of these changes is the point mutation enhancing the promoter of the *ampC* beta-lactamase gene (Table 1). However, this is not the case for the chemostat culture, where no mutations appeared in the same region, implying that adaptation not involving AmpC beta-lactamase can also result in lasting amoxicillin resistance [6].

From a clinical perspective, prolonged or repeated treatment with a single antimicrobial drug may appear to be a poor practice, but this may be common during self-medication, particularly if antimicrobials are easily accessible, as is the case in many countries where antimicrobial drugs are available over the counter [22]. From a scientific point of view, it is also useful to document the effects of repeating or prolonged treatments with the same antibiotic, even if this is clinically less relevant. The outcome of the competition experiments between wild-type cells and the same strain made moderately resistant by a simulated treatment with amoxicillin suggests that the effect of such an initial treatment on a subsequent course is quite dramatic (Figure 3). These findings correspond well with earlier studies [15,23] on the effects of low concentrations of antibiotics on resistance development. When both strains can grow well in separate cultures at the level of antibiotics applied, the mildly resistant strain will take over completely, even if at the start, it is only present as 0.01% of the population. This effect cannot be explained by a difference in growth rates only, and the mechanism behind this rapid take-over is presently not understood. The effect is that the lower boundary of the mutant selection window extends to a level far below the MICs of the susceptible strains, as suggested before on other grounds [13,24]. The effect of exposure to low concentrations of antibiotics is further illustrated by Figure 2, showing that a sub-MIC level of amoxicillin caused the development of resistance by a factor of 16, as the MIC jumped in two days from four to 64 µg/mL. Therefore, it seems that low levels of antibiotics may very well cause great risks of developing resistance.

The supposed fitness costs of antibiotic resistance [14] are not reflected in the competition experiments between the different strains in the absence of antibiotics, as the ratio did not change while the cell density increased by three orders of magnitude. This is in line with the conclusion of a physiological comparison of resistant and susceptible strains, that the price for resistance is not so much metabolic, but rather, a reduced ecological range [6]. It also explains the effect of low concentrations of antibiotic, as a small reduction of the initial growth rate of the sensitive strain (Figure 1) is enough to have a strong influence on the ratio in the co-culture. The observation that the moderately resistant M8 strain outcompetes the sensitive strain more effectively than the highly resistant M256 strain (Figure 3) most likely is not accounted for by metabolic differences between the strains, but by the high beta-lactamase activity of the latter (Table 2). By relatively rapidly clearing the medium from amoxicillin, the M256 strain in fact removes this hurdle for the wild-type cells. Similarly, the equal growth rates of the moderately and highly resistant strains in co-culture can be understood by the elimination of the antibiotic by the beta-lactamase of the M256 strain. A comparable effect might occur in an infection site with a mixture of pathogens with different sensitivities to antibiotics, where at least one pathogen is capable of producing enzymes that lyse antibiotics efficiently.

Both moderate- and high-level resistance to amoxicillin raised the MIC for cefotaxime in the *E. coli* MG1655 variants. Part of this effect may be caused by the induction of high levels of AmpC beta-lactamase, due to mutations in the promoter region in the highly resistant variant [6,8], even though the affinity of this enzyme for cefotaxime is comparatively weak [25]. The effect remains strong enough to lift the MIC in the range of clinically resistant, for the length of a standard antibiotic treatment. After some time, cefotaxime does become effective again, as this antibiotic does not induce AmpC [25] and did not maintain the induction of AmpC (Table 2), but for the outcome of the treatment, this might no longer be relevant. The observed cross-resistance of moderately amoxicillin resistant strains for cefotaxime was not caused by elevated AmpC levels (Table 2), indicating that other cellular processes are involved, as well.

The overall conclusion of this study is that exposure of pathogens to concentrations of antibiotics that fall within the mutant selection window should be avoided as much as possible. Such concentrations can be encountered not only as the result of poor medical practice, but also due to undesirable procedures in agriculture, such as giving antibiotics as a growth-promoter. Both in human medicine and in veterinary practice, considerable thought must be given to which antibiotics are used after treatment failure of the first drug. Using a second antibiotic that has the same or a similar mechanism seems imprudent.

## 4. Experimental

### 4.1. Bacterial Strains, Growth Medium and Culture Conditions

All tested strains were derived from the antibiotic-susceptible wild-type *E. coli* MG1655 strain (WT). The strain, denoted as WT-YFP, which contains the YFP (yellow fluorescent protein) gene and is resistant to chloramphenicol [26], was kindly provided by M. Elowitz. The strains named M8 and M8-YFP were created by growing the WT and the WT-YFP, respectively, at 2 µg/mL amoxicillin for 5 days. These strains became moderately resistant against amoxicillin as a result (MIC 16–32 µg/mL). The strain indicated as M256 was grown at increasing levels of amoxicillin for 2 weeks [5] and had a permanent MIC of 512 µg/mL afterwards. The WT-YFP had the same amoxicillin MIC (4 µg/mL) as the WT, but had an MIC to chloramphenicol of 128 µg/mL.

Batch cultures were grown at 37 °C in the defined minimal mineral Evan’s medium containing 100 mM Na_2_HPO_4_ buffer and 55 mM glucose with a pH of 6.9 [27]. For the cultivation of continuous cultures, the concentrations of glucose and Na_2_HPO_4_ were decreased to 5 mM and 10 mM, respectively. The pH was maintained at 6.9 by pumping 2 N NaOH. The media were autoclaved for 20 min at 121 °C, with the exception of glucose, which was autoclaved for 10 min at 110 °C and added afterwards. Amoxicillin stock solutions of 10 mg/mL were 0.2 mm filter-sterilized and preserved in 4 °C prior to use. 

Precultures for the inoculation of 96-well plates, batch cultures and continuous cultures were grown overnight in 100 mL flasks shaken at 200 rpm at 37 °C. The precultures of susceptible strains (WT, WT-YFP) were grown without antibiotics, while the M8 and M8-YFP precultures were cultivated in medium containing 2 µg/mL amoxicillin and 256 µg/mL for the M256 strain. The experimental concentrations used for simulating suboptimal amoxicillin treatment were 1 or 2 µg/mL. For daily transfers, fresh medium and amoxicillin stocks were used.

Continuous cultures were carried out in Sixfors fermenter vessels (Infors AG, Bottmingen, Switzerland) consisting of 6 vessels with a working volume of 250 mL, at 37 °C and stirred at 250 rpm constantly. The pH of the cultures was regulated at 6.9 by automatically adding the sterile 2 N NaOH. The culture’s parameters, such as pH, temperature and the stirring, were monitored by the controller system of the Sixfors fermenter unit. Amoxicillin treatment was initiated after all the parameters, including the culture’s OD, reached steady state at a dilution rate (D) of 0.3 h^−1^. To mimic the exposure in infection sites as a result of three oral doses per day, amoxicillin was pumped in for an hour, reaching a maximum concentration in the vessel of 2 µg/mL. It was diluted out to approximately 0.3 µg/mL during the subsequent 7 h. The treatment regimen was simulated by repeating these 8-h cycles over 5 days. Samples were taken at exactly 24 h intervals for MIC measurement and sequencing of the promoter of the *ampC* lactamase gene during the entire treatment simulation.

### 4.2. MIC Measurement and Antibiotics

The MIC values were measured in 96-well plates, as described previously [28]. The highest amoxicillin concentration was 1,024 µg/mL with serial dilutions by a factor of 2 until 0.5 µg/mL. The test culture was inoculated to a starting OD600 of 0.05 in the wells. Growth was followed for 23 h by reading OD595 every 10 min with shaking in between and analyzed by the SkanIt software of the Thermo Scientific Multiskan FC (Filter-based Microplate Photometer). All measurements were performed as two technical replicates. The MIC was defined as the minimal concentration of antibiotic that limited growth to an OD of 0.2 or less after 23 h.

### 4.3. Competition Experiment among Susceptible and Resistant Strains

A total of three sets of competition experiments were carried out in this study: WT-YFP and M256, WT-YFP and M8 and M8-YFP and M256. For each experiment, the overnight cultures of the two strains were mixed together at ratios of 1:1, 1:100 and 1:10,000 (resistant:susceptible). The mixed culture was grown in shake flasks containing Evan’s medium either without antibiotics or with 2 µg/mL amoxicillin for 24 h. During the cultivation, the ratio of resistant to susceptible was tracked at *t* = 0, *t* = 3, *t* = 6 and *t* = 24 h by counting colonies from samples on LB (Luria broth). Agar plates containing amoxicillin or chloramphenicol. Plates containing 34 µg/mL chloramphenicol were used to distinguish WT-YFP and M8-YFP strains from their competitors; the plates containing 10 µg/mL or 50 µg/mL amoxicillin were used to select the M8 strain or M256 strain from the mixed cultures; the plates without any antibiotics were used to count the cell numbers of the whole population. Controls showed no growth of susceptible strains on the antibiotic containing plates.

### 4.4. Amplification and Sequencing of the ampC Promoter

The promoter region of the *ampC* gene was amplified by PCR and sequenced using 5'-GGGATCTTTTGTTGCTCT-3' as the forward primer and 5'-CTTCATTGGTCGCGTATT-3' as the reverse primer. Amplification was performed in 50-µL working volumes with Taq DNA polymerase (Thermo Scientific), using the following parameters: denaturation at 95 °C for 5 min, followed by 35 cycles of 35 s at 95 °C, 55 s at 49 °C and 90 s at 72 °C; and finally, 90-s extension at 72 °C. The PCR products were purified with the MSB (Minimal Salt Binding) Spin PCRapace kit (Invitek) and sequenced by Macrogen Europe.

### 4.5. Adaptation of E. coli to Cefotaxime

Three strains were adapted to cefotaxime: WT, M8 and M256. The preculture was initially adapted to the sublethal concentrations: 0.06 µg/mL for WT and M8, 0.5 µg/mL for M256. Whenever normal or approximately normal growth (OD600 > 75% of OD600 for normal growth) occurred, an aliquot of the culture, resulting in an OD600 of 0.1, was used to start two more incubations: one at the same concentration of cefotaxime, the other at double concentration. The stepwise increasing exposure to cefotaxime was continued for 15 days at most. The MIC value was determined every day, and the beta-lactamase activity was measured before and after the adaptation.

### 4.6. Determination of Beta-Lactamase Activity

To measure the activity of beta-lactamase, an assay based on the chromogenic substrate, nitrocefin, was applied [29,30]. Briefly, 1 mL of a culture grown to OD600 of 1.0 was harvested by washing in sodium phosphate buffer (100 mM, pH 7.0). The cells were lysed in sodium phosphate buffer containing 1% Triton X-100, and the cell extracts were centrifuged (15,000 rpm, 5 min, 4 °C). The beta-lactamase activity was determined by measuring the amount of nitrocefin (final concentration: 100 µM) hydrolyzed by 8 µL of the testing sample per minute at 390 nm at 30 °C within 82 µL of sodium phosphate buffer. The final enzyme activity was normalized to the protein concentration of the samples, which was measured using the Thermo Scientific Pierce Micro BCA (Bicinchoninic acid) Protein Assay Kit.

## 5. Conclusions

The overall conclusion from the combined considerations discussed above is that the unsuccessful treatment with one antibiotic can severely hamper a follow-up treatment not only with the same, but also with another, antibiotic of the same class. The exposure to concentrations that simulate those during antibiotic therapy for a similar amount of time led to a consistent and lasting increase in the MIC, severely altering the susceptibility of the strain. A similar conclusion was reached in an *in vivo* situation when studying flock treatment of chickens [31]. Obviously, the best way to prevent these course of events is to render the initial treatment fail-proof by increasing the concentration to a level that ascertains the death of all pathogens. Practical restrictions, such as the tolerance of the patient for the drug, may limit the maximal concentration below the optimal level.
